# Umbilical Hernia Repair and Pregnancy: Before, during, after…

**DOI:** 10.3389/fsurg.2018.00001

**Published:** 2018-01-29

**Authors:** Hakan Kulacoglu

**Affiliations:** ^1^Ankara Hernia Center, Ankara, Turkey

**Keywords:** umbilical hernia, pregnancy, mesh, recurrence, diastasis recti

## Abstract

Umbilical hernias are most common in women than men. Pregnancy may cause herniation or render a preexisting one apparent, because of progressively raised intra-abdominal pressure. The incidence of umbilical hernia among pregnancies is 0.08%. Surgical algorithm for a pregnant woman with a hernia is not thoroughly clear. There is no consensus about the timing of surgery for an umbilical hernia in a woman either who is already pregnant or planning a pregnancy. If the hernia is incarcerated or strangulated at the time of diagnosis, an emergency repair is inevitable. If the hernia is not complicated, but symptomatic an elective repair should be proposed. When the patient has a small and asymptomatic hernia it may be better to postpone the repair until she gives birth. If the hernia is repaired by suture alone, a high risk of recurrence exists during pregnancy. Umbilical hernia repair during pregnancy can be performed with minimal morbidity to the mother and baby. Second trimester is a proper timing for surgery. Asymptomatic hernias can be repaired, following childbirth or at the time of cesarean section (C-section). Elective repair after childbirth is possible as early as postpartum of eighth week. A 1-year interval can give the patient a very smooth convalescence, including hormonal stabilization and return to normal body weight. Moreover, surgery can be postponed for a longer time even after another pregnancy, if the patients would like to have more children. Diastasis recti are very frequent in pregnancy. It may persist in postpartum period. A high recurrence risk is expected in patients with rectus diastasis. This risk is especially high after suture repairs. Mesh repairs should be considered in this situation.

## Introduction

Umbilical hernias are most common in women than men. Pregnancy may cause an umbilical hernia, or render a preexisting one apparent, because of progressively increasing intra-abdominal pressure. Hernia symptoms present in the second trimester in most patients. A hernia may be diagnosed during first, second, or third pregnancies (1). The incidence of an umbilical hernia in pregnant women has been reported to be as low as 0.08% in a very recent large series (2). However, it is possible to meet complicated cases, like a full-term pregnancy in umbilical hernia (3), peritonitis due to skin ulceration (4), or incarcerated pregnant uterus within the hernia rims (5).

A surgical algorithm for a pregnant woman with a hernia is not clear to date, but newer and better scientific data has been cumulated (1, 2, 6). There is no consensus about the timing of surgery for an umbilical hernia in a woman who is already pregnant or planning a pregnancy. In fact, these two types of cases should be taken into consideration separately. Augustin and Majerovic recommended that hernias that are symptomless or have minimal symptoms—including slight discomfort or pain—should be examined regularly and cured electively after delivery and uterine involution (7). Recently, it has been shown that watchful waiting, even up to 5 years, appears to be a safe strategy for ventral hernias in the adult population (8).

It seems to be better and more understandable to stratify the cases into several scenarios regarding the relationship between umbilical hernia and pregnancy. In fact, discussing the issue on a case-by-case basis may be the best approach.

### Umbilical Hernia in Women Planning for a Pregnancy

In this situation, we have several concerns.

- Should we repair the hernia before pregnancy?
- Which repair technique should be used?
- Can the repair remain intact during pregnancy?
- Can the repair cause pain and discomfort during pregnancy?
- How long should the interval between the hernia repair and the pregnancy or birth be?
- What complications can happen during pregnancy if we leave the hernia unrepaired?

**Figure 1:**
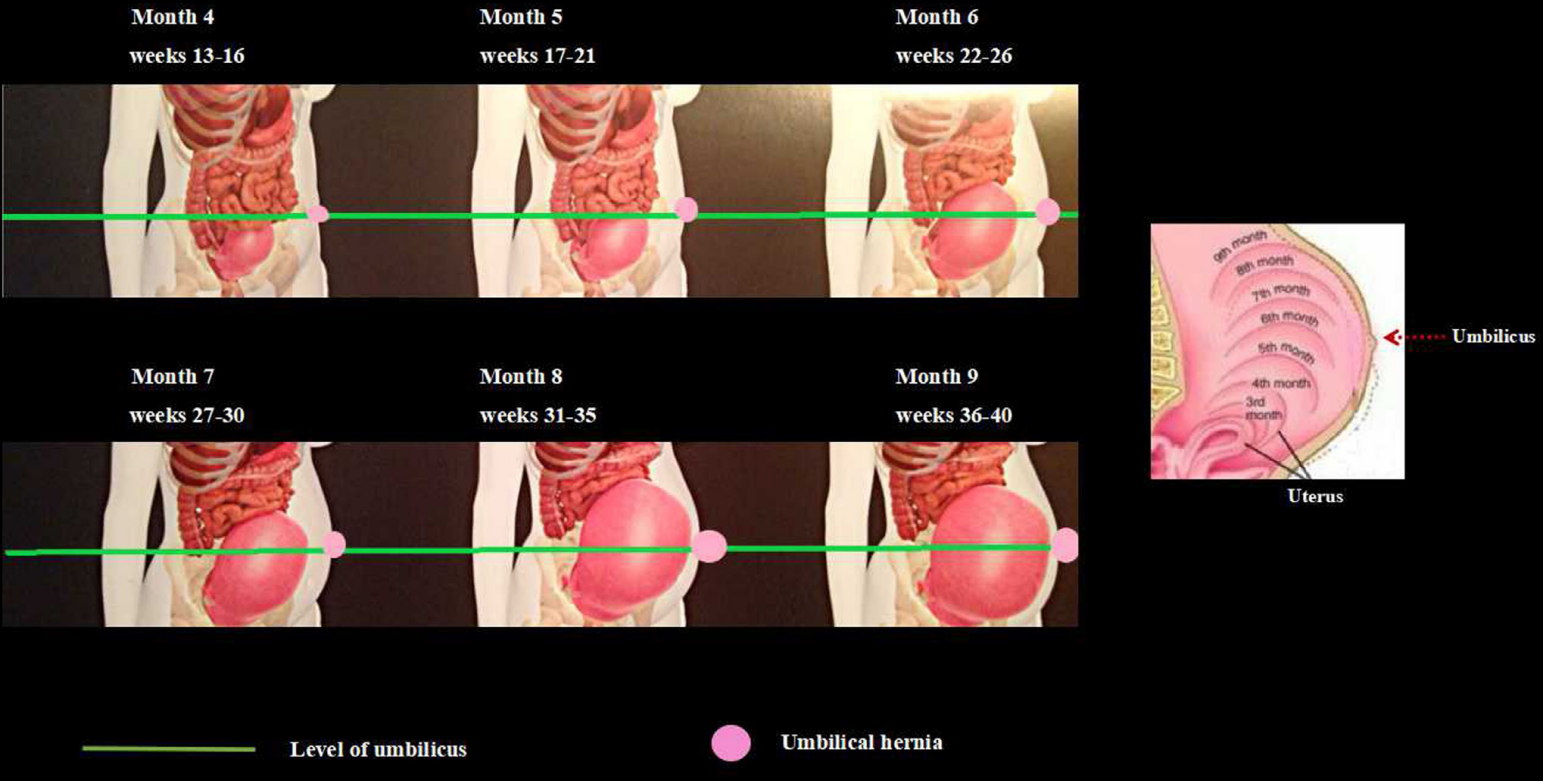
Changes in the size of the uterus and its relation to the umbilicus by the weeks of pregnancy.

When the hernia is incarcerated or strangulated at the time of diagnosis, an emergency repair is inevitable. If the hernia is not complicated, but symptomatic, an elective repair should be proposed. A symptom may be pain or a large bulging. When the patient has a small and asymptomatic hernia, it may be better to postpone the repair until after she gives birth. Fortunately, most of the cases we encounter are in this group. During pregnancy, the enlarged uterus pushes the intestinal loops to superior and posterior parts of the abdominal cavity. The size and pushing force of the uterus during the first trimester does not seem enough to push the intestines into a small umbilical opening. The uterus reaches the level of the umbilicus at about the 20th–22nd week (9, 10). Thereafter, no close contiguity between umbilical hernia defect and intestinal segments exist (Figure 1). If an incarceration occurs during this time, there is less concern about the surgical intervention, because an operation in the first or second trimester would not carry high risks for preterm labor or other adverse effects (11).

A proper repair technique for an umbilical hernia in a woman planning a pregnancy is also a question. It has been shown that mesh repairs provide better outcomes than suture repairs (12). Repairing with only sutures may bring a recurrence during pregnancy (6). Lappen et al. reported that pregnancy caused an increased risk of abdominal hernia recurrence. This information should be given to the patients who are planning an elective hernia repair before a subsequent gestation (13). As the uterus enlarges and intra-abdominal pressure rises, even mesh repairs will not make a pregnant woman immune to hernia recurrence. In concordance, Oma et al. reported that pregnancy after umbilical hernia repair was independently associated with ventral hernia recurrence and mesh use could not lower the risk of recurrence (14). A repair with mesh may restrict the flexibility of the abdominal wall (15) and may cause pain during a subsequent pregnancy (16).

Unfortunately, there is no substantial evidence about the adequate interval between hernia repair and pregnancy or birth. Surgeons usually advise their patients that a pregnancy is not allowed until after the first year of the surgical repair. However, no clinical or experimental studies exist on this specific case. There is no consensus on if this 1-year interval ends at the beginning of the pregnancy or at the time of birth. It can only be said that an early pregnancy may cause recurrence.

**Figure 2:**
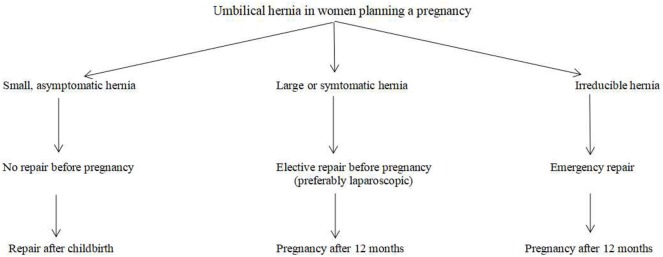
Surgical strategy for umbilical hernia in women planning a pregnancy.

Every hernia carries a risk of incarceration and strangulation. Therefore, patients with an umbilical hernia and planning a gestation should be instructed about this risk. No one can predict which hernias will become complicated or when this will occur. However, every surgeon can tell his or her patient what the malicious effects of an incarcerated or strangulated hernia are on the mother and the baby. An emergency repair, especially during the first or third trimester, will bring the burden of anesthesia and surgical trauma. It should be recommended that patients with large hernias, including intestinal loops, umbilical hernias with a suspicious history of incarceration, and recurrent umbilical hernias previously repaired with a mesh undergo a definitive repair before planning a pregnancy (Figure 2).

### Umbilical Hernia Diagnosed during Pregnancy

**Figure 3:**
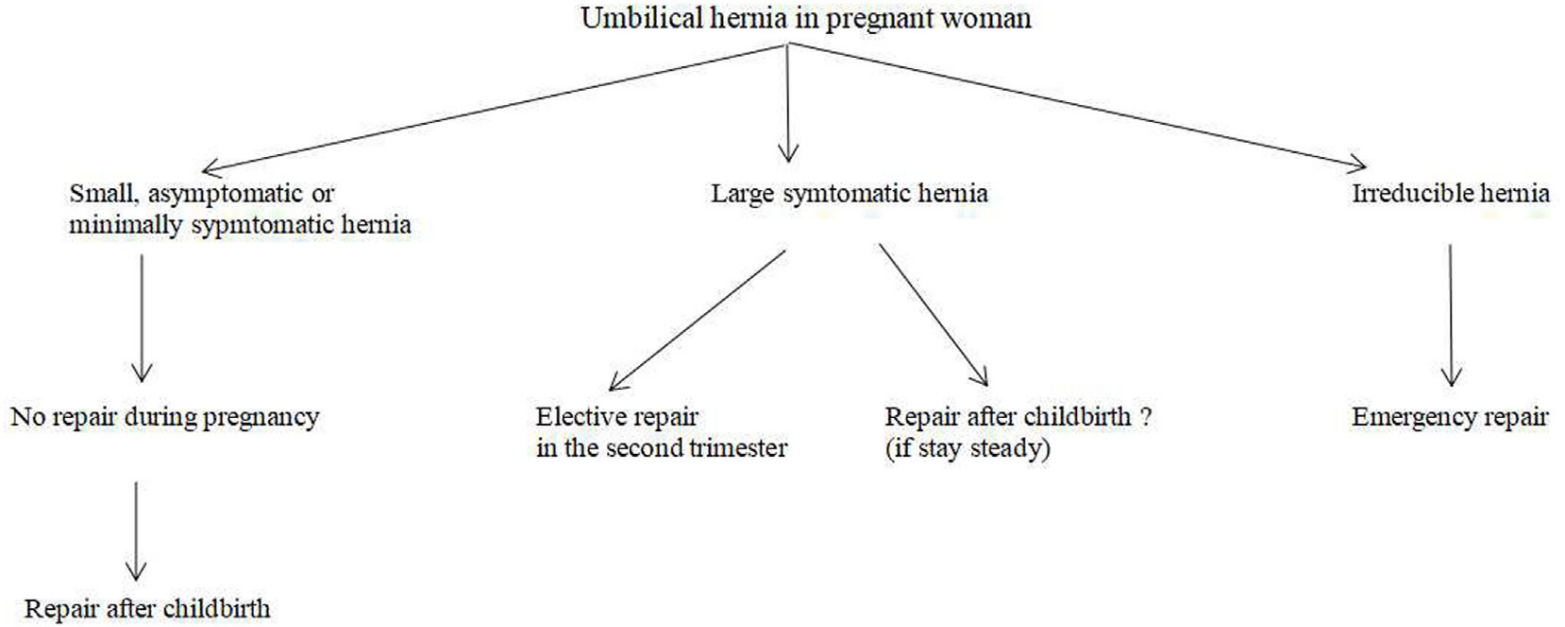
Surgical strategy for an umbilical hernia diagnosed during pregnancy.

Again, there is no solid recommendation for this type of case. Unfortunately, no randomized controlled trial or prospective analysis about hernia repairs in pregnancy existed in the literature (6). However, a small asymptomatic or minimally symptomatic umbilical hernia diagnosed in the early stage of a pregnancy can be managed like a hernia in women planning to become pregnant (Figure 3). Symptomatic umbilical hernias can emerge in every trimester of pregnancy, and they may get incarcerated or strangulated during pregnancy, although the exact rates of these complications have never been reported. Haskins et al. reviewed the American College of Surgeons National Surgical Quality Improvement Program and found that 126 pregnant women were operated on for umbilical hernia repair in a 10-year period (17). Ninety-five percent of the repairs performed with open technique. Incarceration or strangulation existed in half of the cases. Surgery was achieved with minimal 30-day morbidity for the mother and no fetal loss, even in cases of emergencies. Buch diagnosed five female patients with umbilical hernias occurring during pregnancy at the Mount Sinai Medical Center from September 2004 to July 2006 (1). All patients presented with symptoms in the second trimester with reducible hernias. None of them developed incarceration until an open repair following delivery. This finding supports watchful waiting approach during pregnancy (1).

Thirty-one papers, including twenty-three case reports, were found in a recent literature search by Jensen et al. (6). Apart from the above cases mentioned by Haskins and Buch (1, 17), seven patients with an umbilical hernia underwent emergency repair during pregnancy. Suture repair was used in all cases, but one. Wai et al. from Yale University, reported the unique case, describing an intraperitoneal mesh repair for an irreducible umbilical hernia in a woman in the second trimester (18). In Jensen et al.’s literature review, no postoperative complications were recorded (6). This included Ahmed’s emergency repair case with spontaneous rupture in a young, multiparous woman in 28th week of pregnancy (19). In that case, there was skin ulceration due to pressure, and the uterus was completely in the hernia sac with gangrenous intestinal loops of approximately 75 cm. The hernia defect was closed with a suture and the patient gave birth uneventfully 6 weeks later (19).

Oma et al. published the most recent series (2). In this series, 17 pregnant women with an umbilical hernia were recorded within 20,714 pregnancies in a single institution. There were five pregnant patients with an umbilical hernia. Two women noticed the hernia during previous pregnancies, one patient in the present gestation, and the other two at 5th week of pregnancy. All patients completed their pregnancies with no hernia complication.

### Cesarean Section (C-Section) and Simultaneous Hernia Repair

Hernia repair during C-section is a common surgical approach. However, these simultaneous surgeries were not well-documented until the 2000s. In 2004, Ochsenbein-Kölble et al. reported the first case series of C-sections and simultaneous inguinal or umbilical hernia repairs (20). Three patients were offered and underwent combined surgery with their informed consent. In one of them, the sign for C-section was the presence of the umbilical hernia itself. The duration of surgery was longer in cases with inguinal hernia repair, but not umbilical hernia repair versus C-section alone. However, Ghnnam et al. reported that the simultaneous umbilical hernia repair and cesarean needed more time than only a cesarean (21). They compared 48 patients, who underwent cesarean delivery along with paraumbilical hernia repair versus 100 patients undergoing a C-section. Inpatient periods were similar. Only two patients complained of pain at the umbilicus. The control group needed significantly fewer analgesics. Combined surgery was preferred by all patients. One hernia recurred (2.8%), following suture repair within 2 years (21). Mesh repairs were free of recurrence.

Gabriele et al. reported on 28 pregnant women with an inguinal or an umbilical hernia. These patients who underwent simultaneous C-section and hernia repair were compared with 100 patients who only underwent a C-section (22). Combined surgeries took more time for both an umbilical and inguinal hernia than C-section alone. Surgeries were uneventful, and no recurrence developed. The authors concluded that combined surgery is safe and avoids readmissions. Also, Jensen et al. came to a solid conclusion after their literature search that combined hernia repair and C-section is the optimal therapeutic option (6).

Steinemann et al. recently published a retrospective cohort–control study (23). Fourteen patients underwent suture repair of umbilical hernia during C-section by using different techniques. External umbilical hernia repair with suture was used in seven cases *via* a paraumbilical semilunar skin incision after the closure of the Pfannenstiel incision. Internal umbilical hernia repair with suture was used in the other seven patients. Internal suturing required less time than external suturing. Both approaches lengthen the time in operation compared to the control group. Unfortunately, two recurrences were revealed by ultrasonography in each repair subgroup (28%). The authors recommended mesh repairs in these cases (23).

Interestingly, no patient underwent combined surgery in Haskins et al.’s most recent review (17). However, the reason for the absence of any case with simultaneous C-section and hernia repair are not explained in the paper.

### Hernia Repair after Childbirth following an Interval

Some pregnant women with an umbilical hernia do not undergo simultaneous hernia repair at the time of C-section. The reason for that may be a patient’s or surgeon’s choice.

Oma et al. followed eight women with an umbilical hernia and no surgical intervention throughout their pregnancy. The umbilical hernia persisted in all these patients who had a clinical re-evaluation postpartum and no spontaneous disappearance of the hernia was recorded. Elective umbilical hernia repairs were done in five patients within 5 months to 3 years after delivery (2).

Buch et al. reported five cases that underwent hernia repair in the postpartum period. The patients underwent surgery at postpartum for 8–52 weeks. No complications or recurrence were recorded in postoperative follow-up for 2–34 weeks. Two out of five women conceived again after hernia repair. The authors concluded that pregnant patients presenting with reducible groin or umbilical hernias during pregnancy can safely be managed non-operatively during their pregnancy and undergo surgical repair in the postpartum period (1).

Combined surgery may not increase the risk of local and systemic complication (20), however, there are still other concerns about simultaneous surgery. Apart from maternal and fetal health, there are issues regarding the quality and durability of the hernia repair. What would be the advantages of surgical repair in the postpartum period rather than during C-section? In other words, could a concomitant repair during C-section be less reliable? Let us have a look at potential hazards of repair during C-section.

#### Changes in Muscles and Fascial Structures during Pregnancy

The gross structure of rectus abdominis muscle is altered during pregnancy. Significant increases happen in muscle length, separation, and angles of insertions as the pregnancy progressed (24). The functional ability of the abdominal muscles is also altered, and the ability to stabilize the pelvis is decreased. For all abdominal exercises, upper rectus abdominis relative integrated electromyography (EMG) increased while external oblique and lower rectus abdominis relative integrated EMG decreased. Relative EMG for all tested muscles returned to levels seen at 18 weeks and 26 gestations by 18 weeks post-birth. Functional changes found in the rectus abdominis and external and internal obliques. During the immediate post-birth period, separation of the rectus abdominis was resolved by 4 weeks post-birth and abdominal muscle inter-relationships returned to early pregnancy levels by 8 weeks post-birth. However, the ability to stabilize the pelvis remained low at 8 weeks post-birth. This sustained decrement in the ability to stabilize the pelvis at 8 weeks post-birth may reflect the poor resolution of abdominal muscle length increases due to pregnancy (24).

In fact, the meaning of the alterations in abdominal muscle groups for the fate of an umbilical hernia repair is obscure. Whether the changes increase or decrease, hernia recurrence rates is unknown to surgeons. However, the abdominal muscles during pregnancy differ from usual. It may be better to wait for a while to let the muscles return to their normal anatomy and function before repairing the umbilical hernia. However, there is no recommendation in the literature for the exact time to wait for a repair.

#### Relaxin. Is it ImWportant?

Another issue that may affect the fate of hernia repair in a pregnant or early postpartum woman is hormonal changes during gestation. Relaxin is a peptide hormone in the insulin family, secreted by the corpus luteum (25). It is also released from the placenta during pregnancy. It relaxes pelvic ligaments and softens and widens the cervix. Relaxin reduces extracellular matrix (ECM) synthesis and induces collagen degradation (26). In a study on rats, relaxin caused a significant reduction in tissue collagen content (27). Relaxin limited collagen production, while stimulating increased collagen degradation (28). Also, Naqvi et al. documented relaxin’s degradative effects on joint fibrocartilaginous tissue with matrix degradation by metalloproteinases (MMPs) (29).

Collagen, ECM, and MMPs have important implications for hernia formation. Collagen is the most abundant ECM protein. Collagenase, a member of the MMP family, is the principal enzyme in collagen degradation (30).

Considering the studies on the relationship between collagen, ECM, and MMPs, we can think any endogenous or exogenous substance that affects these mechanisms may cause recurrence after hernia repair, especially following suture repairs. Therefore, we can say there may be a risk of recurrence when the repair is done and the relaxin level is high. Although there is no evidence for this supposition, there are interesting reports in the literature. It has been reported that a higher expression of relaxin receptors within the muscles of the pelvic diaphragm in dogs with a perineal hernia. This may suggest that relaxin plays a role in the pathogenesis of this type of hernia by causing muscular atrophy (31). Relaxin may also be a factor in perineal hernia formation with connective tissue degeneration in dogs (32). In human beings, there is only one report on the relation between relaxin and abdominal hernias (33). In this study, all the children born in Malmö, Sweden in a 5-year period were checked for congenital dislocation of the hip (CDH) and for an inguinal hernia. Hernia was diagnosed five times more frequently in girls with CDH than girls without, and three times in boys with CDH than boys without. The authors stated that relaxin could stimulate collagenase, induce structural changes in the connective tissue, and cause development of both CDH and the hernia (33). This paper was published in 1988 and no further data on the subject has been collected since.

#### Would Lifting and Carrying Baby Create a Burden on the Repair?

Surgeons generally put patients on a weight lifting restriction after hernia repairs. Even mesh repairs are vulnerable to rises in intra-abdominal pressure in the early postoperative period. Biomechanical studies have revealed that the tensile strength provided by tissue ingrowth into the mesh reaches approximately 80% after only 6 weeks (34). Although there is no consensus on weight lifting restriction after hernia repairs, surgeons do not want their patients to lift any weight for the first 2 weeks. Moderate lifting (<10 kg) is allowed after 2–4 weeks. Patients are advised to lift over 10 kg only after 8 weeks (35). In fact, carrying and lifting a baby would stay within the limits of the advice. However, a woman who does not have a baby and undergoes umbilical hernia repair would be on a weightlifting restriction for a much longer time.

Although umbilical hernia repair can be performed after childbirth, there is no need for surgery on small asymptomatic hernias in the early postpartum period. A 1-year interval can give the patient a very smooth convalescence, including hormonal stabilization and return to normal body weight. Surgery can be postponed for a longer time, even after another pregnancy, if the patient would like to have more children.

### Significance of the Concomitant Diastasis Recti (DR)

Diastasis recti is the midline separation of the rectus abdominis muscles. It is an impairment, but not a true hernia, and does not carry a risk for incarceration. There is a positive correlation between parity and DR (36). The prevalence during pregnancy is about 30–70%. The normal width of the linea alba is 15 mm at the level of xiphoid, 22 mm at the level of 3 cm cranial to the umbilicus, and 16 mm at the level of 3 cm caudal to the umbilicus in nulliparous women (37). Mechanical forces and hormonal changes during pregnancy may play a role in the etiology.

The most frequent localization is in the periumbilical region and persistence postpartum is found in about 60% of cases (38). Liaw et al. reported that diastasis may persist in the postpartum period and the abdominal muscle function improved, but did not return to normal, even after 6 months (39). Sperstad et al. followed 300 first-time pregnant women from pregnancy until 12 months postpartum. They reported that DR existed in 33.1, 60.0, 45.4, and 32.6% of the women at 21 weeks of pregnancy, and at 6 weeks, 6 months, and 12 months following delivery, respectively (40). This study revealed that the risk for DR was twofold higher in women reporting heavy lifting 20 times a week or more than in women reporting less weight lifting. The authors did not describe the heavy lifting in the text, but we can assume that a postpartum woman lifts her baby many times a week. The weight of a baby is about 8 kg at 6 months and 10 kg at 12 months (41). These weights are enough to raise intra-abdominal pressure as high as a Valsalva maneuver does (35).

Although RD is not a hernia, it may cause recurrence as a larger hernia following umbilical hernia repairs. In umbilical hernia repairs with sutures, the bites pass through a weak rectus sheet at the region of diastasis. This may cause tears and create button hole defects, consequently resulting in recurrence. Köhler et al. evaluated 231 suture repairs for small primary umbilical or epigastric hernias (42). Hernia defects were smaller than 2 cm. Patients with rectus diastasis developed hernia recurrence at a significantly increased rate. The authors hypothesized that thin and stretched rectus sheath is a risk factor for recurrence. They recommended mesh repair for umbilical hernia patients with rectus diastasis. Although Emanuelsson et al.’s recent prospective randomized study showed that two-row suture plication with delayed absorbable material provided similarly good results with retromuscular lightweight polypropylene mesh without an increase in recurrence rate in treatment of RD (43), mesh use remains a better option for patients with concomitant umbilical hernia and RD (42). In addition, one can assume that a recurrence still may develop from the sites of mesh fixation if there is a vulnerable linea alba due to RD. Therefore, it is better to use no fixation in case of strong restoration of the line alba or to use an autraumatic mesh fixation like glues (e.g., fibrin) (44) or a self-gripping mesh in retromuscular mesh repairs (45).

## Conclusion

There is no consensus about the timing of surgery for an umbilical hernia in a woman who is already pregnant or planning a pregnancy. If the hernia is incarcerated or strangulated at the time of diagnosis, an emergency repair is inevitable. If the hernia is not complicated, but symptomatic, an elective repair should be proposed. If the hernia is repaired by suture, the risk of recurrence is high during pregnancy. Repair with a mesh may restrict the flexibility of the abdominal wall and may cause pain during a subsequent pregnancy. When the patient has a small and asymptomatic hernia, it may be better to postpone the repair until she gives birth.

Umbilical hernia repair during pregnancy can be performed with minimal morbidity to the mother and no fetal loss even in emergency cases. If a small hernia becomes larger and symptomatic, the second trimester is a proper period for surgery. Umbilical hernias can be repaired following childbirth or at the time of C-section. Patient satisfaction is high for combined C-section and hernia repair. However, a high recurrence rate is expected.

Elective repair after childbirth is well-documented. It is possible as early as the postpartum at 8 weeks. There is no need for surgery for small asymptomatic hernias in the early postpartum period. A 1-year interval can give the patient a very smooth convalescence, including hormonal stabilization and return to normal body weight. Surgery can be postponed for a longer time, even after another pregnancy, if the patient would like to have more children.

Diastasis recti are very frequent during pregnancy. It may persist in the postpartum period. Patients with rectus diastasis may develop umbilical hernia recurrence after repair. This risk is especially high following suture repairs. Mesh repairs should be considered in this situation (Table 1).

**Table 1:** Pros and cons for specific conditions in the relation of umbilical hernia and pregnancy.

	Suture repair	Mesh repair	
Umbilical hernia in woman planning a new baby	High risk of recurrence	Pain in third trimester	Repair is postponed until birth for small and asymptomatic hernias
Umbilical hernia diagnosed during pregnancy	High risk of recurrence	Infection risk for pregnant woman especially in emergency repairs	Repair is postponed until birth for small and asymptomatic hernias
Cesarean section and simultaneous hernia repair	Easier	Requires separate incision	Patient satisfaction can be high
	May be performed without separate incision	Lengthen operative time	Patient’s preference should be asked
	High risk of recurrence	Infection risk in puerperium	
Hernia repair after childbirth	No exact recommendation for timing	No exact recommendation for timing	A 1-year interval may be recommended
			Repair can be postponed for another pregnancy
Concomitant diastasis recti	High risk of recurrence	Recommended	Patient should be informed about diastasis

## Author Contributions

The author confirms being the sole contributor of this work and approved it for publication.

## Conflict of Interest Statement

The author declares that the research was conducted in the absence of any commercial or financial relationships that could be construed as a potential conflict of interest.
